# Supplementary material for: Complete Revascularization and One-Year Survival with Good Neurological Outcome in Patients Resuscitated from an Out-of-Hospital Cardiac Arrest
Source: J Clin Med. 2022 Aug 29;11(17):5071. doi: 10.3390/jcm11175071 (PMC9456720; doi:10.3390/jcm11175071)
Supplement: Supplementary file 1 [file jcm-11-05071-s001.zip › jcm-1821808-supplementary.pdf]

Supplementary Table S1. Characteristics of resuscitated OHCA patients with multivessel disease.

		(n=119)
**Age (years)**		67 ± 12
**Sex**		
	**Male**	98 (82.4%)
	**Female**	21 (17.6%)
**BMI (kg/m2)**		26 ± 4.4
**Hypertension**		90 (81.1%)
**Diabetes**		19 (17.3%)
**Smoker**		35 (31.5%)
**Hypercholesterolemia**		67 (60.4%)
**Previous myocardial revascularisation**		
	**Previous PCI**	18 (16.1%)
	**Previous CABG**	8 (7.1%)
**Rhythm at presentation**		
	**Shockable**	100 (84%)
	**Not shockable**	19 (16%)
**First rhythm detected**		
	**VF**	88 (73.9%)
	**Pulseless VT**	3 (2.5%)
	**PEA**	13 (10.9%)
	**Asystole**	5 (4.2%)
	**AED shockable**	9 (7.6%)
	**AED not shockable**	1 (0.8%)
**ECG diagnostic for STEMI**		75 (74.3%)
**HR (bpm)**		101 ± 30
**Median CA duration (min)**		23 (18.7-29.9)
**Time from CA to CAG (days)**		0.7 ± 2.4
**LVEF (%)**		40 (37-40)
**Pharmacological haemodynamic support (%)**		37 (33.3%)
**IABP**		9 (8.2%)
**ECMO**		7 (6.4%)
**IABP+ECMO**		6 (5.5%)
**Serum Creatinine at admission (mg/dl)**		1.03 (0.9-1.1)
**hs-TNI at admission (ng/l)**		913 (585-1862)
**hs-TNI peak value (ng/l)**		52611 (21216 – 73783)
**CK at admission (U/L)**		263 (220 – 341)
**CK peak value (U/L)**		2106 (1620 – 2500)
**Hb at admission (g/dl)**		13.4 ± 1.9
**Survival at discharge**		73 (61.9%)

AED, automated external defibrillator; BMI, body mass index; CABG, coronary artery by-pass graft; PCI, percutaneous coronary intervention; VF, ventricular fibrillation; VT , ventricular tachycardia; PEA, pulseless electrical activity; ECG, electrocardiogram, STEMI, ST-elevation myocardial infarction; HR, heart rate; CA, cardiac arrest; CAG, coronary angiography; LVEF, left ventricular ejection fraction; IABP, intra-aortic balloon pump; ECMO, extra-corporeal membrane oxygenation; hs-TNI, high-sensitivity cardiac troponin I; CK, creatin-kinase; Hb, haemoglobin.

Supplementary Table S2. Characteristics of resuscitated OHCA patients with multivessel disease discharged alive.

	(n=73)
**Age (years)**	66.3 ± 12.3
**Sex**	
**Male**	62 (84.9%)
**Female**	11 (15.1%)
**BMI (kg/m2)**	25.8 ± 4.8
**Hypertension**	57 (80.3%)
**Diabetes**	12 (16.9%)
**Smoker**	27 (38.0%)
**Hypercholesterolemia**	47 (66.2%)
**Previous myocardial revascularisation**	
**Previous PCI**	9 (12.7%)
**Previous CABG**	6 (8.5%)
**Rhythm at presentation**	
**Shockable**	68 (93.2%)
**Not shockable**	5 (6.8%)
**First rhythm detected**	
**VF**	58 (79.5%)
**Pulseless VT**	3 (4.1%)
**PEA**	3 (4.1%)
**Asystole**	2 (2.7%)
**AED shockable**	7 (9.6%)
**AED not shockable**	0 (0%)
**ECG diagnostic for STEMI**	46 (71.9%)
**HR (bpm)**	102.0 ± 31.0
**Median CA duration (min)**	18.0 (13.8 - 22.1)
**Time from CA to CAG (days)**	1.0 ± 2.97
**LVEF (%)**	40.0 (40.0 - 45.0)
**Pharmacological haemodynamic support (%)**	12 (17.4%)
**IABP**	3 (4.4%)
**ECMO**	0 (0%)
**IABP+ECMO**	3 (4.4%)
**Serum Creatinine at admission (mg/dl)**	0.96 (0.90 - 1.03)
**hs-TNI at admission (ng/l)**	457 (306 - 913)
**hs-TNI peak value (ng/l)**	47530 (12888 - 63240)

**CK at admission (U/L)**	170 (142 - 262)
**CK peak value (U/L)**	1768 (1121 - 2401)
**Hb at admission (g/dl)**	14.0 ± 1.8

AED, automated external defibrillator; BMI, body mass index; CABG, coronary artery by-pass graft; PCI, percutaneous coronary intervention; VF, ventricular fibrillation; VT , ventricular tachycardia; PEA, pulseless electrical activity; ECG, electrocardiogram, STEMI, ST-elevation myocardial infarction; HR, heart rate; CA, cardiac arrest; CAG, coronary angiography; LVEF, left ventricular ejection fraction; IABP, intra-aortic balloon pump; ECMO, extra-corporeal membrane oxygenation; hs-TNI, high-sensitivity cardiac troponin I; CK, creatin-kinase; Hb, haemoglobin.
